# Novel embryo selection strategies—finding the right balance

**DOI:** 10.3389/frph.2023.1287621

**Published:** 2023-12-15

**Authors:** Alex Polyakov, Genia Rozen, Chris Gyngell, Julian Savulescu

**Affiliations:** ^1^ Faculty of Medicine and Health Sciences, The University of Melbourne, Parkville, VIC, Australia; ^2^ Reproductive Biology Unit, Royal Women’s Hospital, Melbourne, VIC, Australia; ^3^ Melbourne IVF, Melbourne, VIC, Australia; ^4^ Murdoch Childrens Research Institute, Royal Children’s Hospital, Melbourne, VIC, Australia; ^5^ Centre for Biomedical Ethics, Yong Loo Lin School of Medicine, National University of Singapore, Singapore, Singapore

**Keywords:** infertility, embryo, novel technologies, IVF (*in vitro* fertilization), PGT (preimplantation genetic testing)

## Abstract

The use of novel technologies in the selection of embryos during *in vitro* fertilisation (IVF) has the potential to improve the chances of pregnancy and birth of a healthy child. However, it is important to be aware of the potential risks and unintended consequences that may arise from the premature implementation of these technologies. This article discusses the ethical considerations surrounding the use of novel embryo selection technologies in IVF, including the growing uptake of genetic testing and others, and argues that prioritising embryos for transfer using these technologies is acceptable, but discarding embryos based on unproven advances is not. Several historical examples are provided, which demonstrate possible harms, where the overall chance of pregnancy may have been reduced, and some patients may have missed out on biological parenthood altogether. We emphasise the need for caution and a balanced approach to ensure that the benefits of these technologies outweigh any potential harm. We also highlight the primacy of patients' autonomy in reproductive decision-making, especially when information gained by utilising novel technologies is imprecise.

## Introduction

During *in vitro* fertilisation, oocytes and sperm are combined to produce an embryo. In a typical stimulated cycle, more than one embryo is available for transfer. The traditional approach has been to transfer the “best” embryo first. “The best” is conventionally defined as an embryo with the highest potential to result in a viable pregnancy (1). Numerous developments in reproductive medicine attempt to improve embryo selection to achieve pregnancy sooner. These include the application of artificial intelligence (AI), embryo genetic testing for aneuploidy, and time-lapse analysis, to name a few. Some novel technologies even go further and promise to select an embryo with the healthiest possible future life, such as embryo selection based on the Polygenic Risk Score analysis (2). While avoidance of disease and embryo selection to achieve the healthiest possible offspring in the shortest possible time are worthy goals, one must also be acutely cognizant of the potential of these novel technologies to cause harm, primarily when they are used to deselect and discard embryos. The concept of non-maleficence must be balanced against the principle of maximising expected utility, where the overall benefit outweighs possible harm to individual patients, resulting in overall benefit.

Unfortunately, there are numerous instances where widespread premature implementation of novel embryo testing and selection strategies resulted in a possible decreased chance of achieving a viable pregnancy. It is likely that on some occasions, patients even missed out on having biological offspring altogether. Researchers and clinicians must continue to strive to help patients achieve a viable pregnancy and birth of a healthy child in the shortest possible time frame. At the same time, they must ensure that their efforts do not decrease the overall chance of pregnancy. We propose that relying on novel technologies to prioritise embryo transfer is ethically acceptable, but discarding embryos based on these unproven technological advances is not. In other words, we must strive against overconfidence to ensure that novel technologies do not result in unintentional overall harm.

## Ethics of selecting an embryo

It is common to have several viable embryos available for transfer at the end of an IVF cycle. There has been a gradual and welcome shift to transfer one embryo at a time to minimise the risks of multiple pregnancies (3) without compromising the overall chance of success (4). Therefore, a decision must be made about which embryo will be transferred first, second, etc. The extensive philosophical discourse on the non-identity problem is beyond the scope of this article. It is sufficient to say that we take it as a given that embryos cannot be harmed in a conventional sense if they are either transferred later or not transferred at all. The question then arises as to what are the morally acceptable reasons to select one embryo in preference to another? There appear to be two rational starting points on which to base the choice: the shortest possible time to pregnancy and the prevention of children being born with conditions that will significantly adversely affect the length or quality of their life. It must be acknowledged that the second criterion, namely the prevention of a birth of a child with a disability, is controversial (5, 6). Nevertheless, it is widely accepted in the community, among health professionals and ethicists (7–10).

We propose a third criterion that must be considered, especially when incorporating novel and unproven methods of embryo selection—the overall chance of pregnancy from a stimulated IVF cycle (which can be termed “cumulative live birth rate per stimulated cycle started”) must not be reduced by a novel selection strategy, compared to the currently accepted selection/deselection criteria. This implies that when novel selection criteria are implemented, the overriding principle should be to only select embryos for transfer but not to discard embryos. It must be clearly recognised that a discarded embryo has a zero chance of producing a pregnancy, while a poor-quality embryo, deemed non-viable by a novel test, may still have a chance of producing a healthy baby (11, 12). After all, the nature of any test includes false positive and false negative outcomes, and no test is 100% accurate. We will now provide several examples where novel technologies were implemented without appropriate evaluation, which most likely resulted in an overall reduction in the chance of pregnancy per stimulated cycle started, depriving some patients of genetically related offspring.

## Pre-Implantation genetic screening (PGS)

It is generally accepted that some embryos created by IVF may be chromosomally abnormal. Transferring such embryos will either result in implantation failure, miscarriage, or the birth of a child with a significant disability, such as trisomy 21 (Down syndrome). It follows that if the chromosomal complement of an embryo can be ascertained before an embryo transfer, only chromosomally normal embryos can be utilised, thus reducing or even eliminating the risk of these undesired outcomes. Initial proof-of-concept studies were performed in the early 1990s, demonstrating the possibility of removing a small number of cells from an early embryo for genetic analysis (13) for medically indicated sex selection. This was followed by the introduction of fluorescence *in situ* hybridisation (FISH), which allowed the assessment of multiple numerical chromosomal aberrations simultaneously (14, 15). This technology was widely used where cleavage stage embryos (day2–3 after fertilisation) were biopsied, and embryos that produced an abnormal result were discarded.

This widespread dissemination and utilisation of this novel embryo selection technology were based on several assumptions that were subsequently demonstrated to be false. For example, it was widely assumed that the implantation potential of an embryo is not affected by the biopsy process. Eventually, in 2007, the first Randomized Controlled Trial (RCT) was published comparing PGS with standard IVF. It demonstrated a reduction in live births from 35% in conventional IVF to 24% in patients where PGS was used (16). This finding was attributed to embryo damage due to the biopsy (17) and the possible high prevalence of false positive results (wrongly labelling chromosomally normal embryos as abnormal), confirmed by further studies (18). It is undeniably true that numerous patients were harmed by the premature introduction and widespread uptake of this technology, as a consequence of damaging and wrongly discarding embryos with normal reproductive potential.

The technique of cleavage-stage embryo biopsy and FISH PGS is now largely abandoned, only to be replaced by what is termed PGS 2.0 (or PGT-A), where blastocyst embryos (day 5/6) are biopsied and comprehensive genetic screening utilising Next Generation Sequencing (NGS) technology replacing FISH. Despite the fact that numerous RCTs and large retrospective studies demonstrated its limited effectiveness and possible reduction in pregnancy rate per cycle started (19–22), this iteration of pre-implantation genetic testing is being utilised worldwide with increasing frequency (23). PGS 2.0, similar to the originally used PGS by FISH, lacks scientific support (24–27) and may be responsible for falling IVF success rates in countries with high utilisation rates (28, 29). There possibly exist some groups of patients who may benefit from this technology, such as women affected by recurrent implantation failure (30, 31), but even this indication is highly controversial (32), since the sub-group of women in question is ill-defined (33, 34). There are also well-described technical limitations of PGT-A, such as allele dropout, which may render this technique even less reliable than originally thought (35, 36).

It must be acknowledged that numerous published reports, at first glance, appear to support both limited and widespread use of PGT-A in terms of several clinically relevant outcomes. For example, Neal et al. (2018) modelling study concluded that “For patients with > 1 embryo, IVF with PGT-A reduces healthcare costs, shortens treatment time, and reduces the risk of failed embryo transfer and clinical miscarriage when compared to IVF alone.” (37) The same issue of the Journal published an opinion that questioned the assumptions of this model and its conclusions (38). This opinion piece echoed concerns regarding widespread PGT-A use outlined above and pointed to significant limitations of the modelling approaches to the critical evaluations of the utility of PGT-A. Regarding cost-effectiveness, the published data is inconclusive and contradictory, demonstrating benefit in some studies in specified patient groups (39, 40) but not in others (41, 42).

Another retrospective study supported the utility of PGT-A and its positive impact on live birth (Sanders et al., 2021) (43). A different analysis of the same cohort was highly critical of the methodology used. It came to a radically different conclusion, where the utilisation of PGT-A resulted in a modest but significant reduction in cumulative Live Birth Rate (OR = 0.82) (44).

The study by Tiegs et al., 2021 utilised PGT-A but all embryos, irrespective of the ploidy status, were transferred prior to the results of the biopsy being known. While it demonstrated the analytical utility of PGT-A in terms of zero diagnostic clinical error rate, it also produced equivalent sustained implantation between the study group and an age-matched control group, where a biopsy was not performed (47.9% vs. 45.8) (45). This particular study did not address the clinical utility of PGT-A, but rather the test's analytical validity. The accompanying editorial also raised the issue of some embryos destined for genetic testing being discarded due to inconclusive results in clinical practice, thus reducing the cumulative pregnancy rate per stimulated cycle started (46).

Another large retrospective cohort study based on the data from Society for Assisted Reproductive Technology Clinic Outcome Reporting System (SART) collected between 2014 and 2018 came to the following conclusion: “Cumulative LBRs were significantly lower in cycles that used PGT than those that did not among women younger than 35 years, regardless of the number of oocytes retrieved, but did not significantly differ by the use of PGT among older women.” (47) It must be noted that 8% of patients who elected to utilise PGT-A in this cohort had no viable embryo available for transfer. The chance of pregnancy for those patients was zero, but their outcomes are not included in the reported data.

These are just some examples of scientific reports that may support the use of PGT-A but, on close inspection, are consistent with the conclusion that the overall utility of this technique is highly debatable. It is also often argued that selecting only euploid embryos for transfer shortens the time to pregnancy and reduces the risk of miscarriage. Unfortunately, none of the studies available at present support these assertions (19, 48, 49).

Where PGS is employed to prevent disability, it must be compared to prenatal testing in terms of its chance of reducing pregnancy overall and damaging the embryo, potentially causing harm to the future child. While PGS avoids the need for the termination of pregnancy of affected fetuses, it does have potential downsides both for the woman (in terms of potentially reducing the overall chance of pregnancy from the IVF cycle) and damaging the embryo, resulting in harm to the future child. Women/couples should be informed of these drawbacks.

## Non-invasive genetic testing and selection

It is intuitive that extracting cells from a developing embryo is not without risks. The most obvious concern is embryo damage which may render an embryo unsuitable for transfer or freezing. Such an embryo, which is discarded, has a zero chance of producing a viable pregnancy, while if it was transferred without biopsy, even if it were of low quality, would have a chance of becoming a baby. Furthermore, perhaps more importantly, it is largely unknown if a trophectoderm biopsy adversely impacts the health and well-being of future offspring (50). The impact and the risk of late-onset diseases will not be known for decades, but there are reports of increased risk of some pregnancy-related complications, particularly hypertensive disorders of pregnancy, attributable solely to a trophectoderm biopsy (51, 52). These reports are retrospective and may be subject to numerous confounders and biases. Therefore, their conclusions must be treated with caution and require further investigation. Nevertheless, it is generally agreed that avoiding an embryo biopsy would be desirable. Hence, various technological solutions have been sought to ascertain embryos' genetic complements in a non-invasive fashion, i.e., without a biopsy. These commonly rely on an embryo reaching certain developmental milestones or some other characteristics (53–56), commonly interpreted using Artificial Intelligence (57–59). Crucially, in the past, such technologies were only ever used for embryo selection/prioritisation, and even embryos considered to be suboptimal were eventually transferred once embryos assessed as being “better” were used up without success.

One incident deserves special consideration as it provides the clearest example of premature untested technology being used not only to prioritise embryos for transfer but also to possibly discard them, reducing the overall chance of pregnancy for patients and depriving some of the opportunity of biological parenthood. Embryo culture media contains DNA derived from discarded embryonic cells (60). Over the past decade, numerous attempts have been made to analyse this extra-embryonic DNA to ascertain the reproductive potential of embryos (61, 62). This work is ongoing (63), and up until recently, this technology was unavailable for clinical use outside research protocols.

In May 2019, a large IVF clinic in Victoria, Australia, announced a scientific breakthrough (64, 65). Described as a genetic test of the fluid an embryo is cultured in, it was reported to be based on two clinical trials lasting two years. It was offered and promoted to patients at the cost of AU$495 (66). The aforementioned trials were never published or even presented at a conference. The National Association of Testing Authorities (NATA) accredited the test as being consistent with contemporary laboratory practices. Crucially, NATA does not validate a test's clinical use or accuracy but only assesses its laboratory performance. The test was widely used by an unknown number of patients, and embryos classified as genetically abnormal based on this test were reportedly discarded. In October 2020, the Therapeutics Goods Administration (TGA, Australian Food and Drug Administration equivalent) received a report from the IVF clinic, notifying it of a discordancy between validation studies (that were never published and are not in the public domain) and clinical experience with the test (67). From this report, it can be ascertained that the novel non-invasive test produced an unexpectedly high proportion of abnormal results, an order of magnitude higher than a more established commonly used technique which involved an embryo biopsy. This outcome can also be clearly seen in the Victorian Assisted Reproductive Treatment Authority (VARTA) 2021 Annual Report (Table 1.6) (68).

In the financial year 2019–2020 (July 2019 to June 2020), the IVF clinic performed conventional PGT-A on 568 embryos, 313 of which were deemed suitable for transfer, a rate of 55.1%. In this time period and in the same laboratory, 977 embryos were tested with the novel non-invasive PGS of the culture fluid. Only 284 embryos were assessed as genetically normal and suitable for transfer, a rate of 29.1%. This implies that 44.9% of embryos were classified as abnormal and discarded using the conventional embryo biopsy-based test vs. 70.9% being classified as abnormal and possibly discarded, based on the finding of the non-invasive PGT-A testing. Furthermore, in the preceding year, 239 women treated with the conventional PGT-A gave birth to a total of 115 babies, at the rate of 48.2 babies per 100 women treated. On the other hand, the non-invasive test was used for 91 women who gave birth to 33 babies, a much lower birth rate of 36.3 babies per 100 women treated. Most importantly and significantly, embryos deemed genetically abnormal and, therefore, unsuitable for transfer appear to have been discarded in both groups. These developments were widely reported in the Australian media (69–71) and are currently the subject of a class action litigation in Victoria, Australia (72). It is alleged that some patients did not achieve pregnancy due to the utilisation of the novel non-invasive PGS, and some missed out on having genetically related offspring.

The above data from the VARTA report speak for itself and broadly supports this conclusion. There is also an extensive body of evidence to support the contention that developing embryos may preferentially expel genetically abnormal DNA, may be contaminated with maternal DNA, and that the obtained samples of culture fluid may be uninterpretable due to high levels of embryonic mosaicism (63, 73). These aspects of non-invasive PGS could explain the results obtained by the IVF clinic and can also illuminate the extent of these problems with the non-invasive testing techniques.

Overall, it must be concluded that the major error made by introducing this new technology without appropriate evaluation was that embryos deemed abnormal by the novel test were probably discarded. This appears to be a case of a premature introduction of a novel technology into clinical practice, outside well-regulated research protocols, without prior publication of validated studies and the clinic potentially deriving financial benefit from this endeavour by charging patients for an unproven test. An unacceptable and surprisingly high false positive rate was only apparent when enough patients had undergone the procedure. An appropriately designed trial would have prioritised patient safety and clinical effectiveness and should have included the storage of embryos deemed genetically abnormal and, therefore, unsuitable for transfer. The cost of storing extra embryos should have been borne by the clinic conducting the trial. Further testing with a conventional technique or transfer without genetic testing would have been an option for patients participating in such a trial. It would have prevented any loss of embryos, resulting in a non-inferior pregnancy rate per IVF cycle initiated. Furthermore, when the incidence of aneuploidy in both conventional and non-invasive PGS groups is taken into account (45% vs. 71%), a simple sample-size calculation demonstrates that only 55 embryos tested in each group would have been sufficient to detect such a difference (74). The ethics of rushing an unproven technology into unrestricted clinical use is beyond the scope of this paper, but it is ethically unacceptable for a clinic to attempt to gain a competitive advantage and financial gain at the expense of patients' clinical outcomes.

## The right to choose

One uncontroversial statement can be made about a cohort of embryos produced from an IVF cycle: some will result in the birth of a child, while others will not. There is no universally accepted technique to differentiate one group from the other. It is beneficial to aim to achieve a viable pregnancy and a healthy child in the shortest possible time, and therefore any advancement that would select an embryo with the highest reproductive potential and to prioritise such an embryo for transfer in preference to “worse” embryos has merit. The issue that has arisen time and time again is that most selection technologies rely on embryo manipulation, which may damage an embryo, reducing the overall success rate of an IVF cycle. It is also worth noting that any test has inherent limitations with false positive and false negative rates, which may classify some embryos as “abnormal” when they may have a normal reproductive potential.

New generation technologies aim to select an embryo with the highest chance of producing a healthy child without embryo manipulation, avoiding possible damage. These include the use of AI, developmental morphological characteristics analysis, and culture fluid evaluation, to name a few. These tests, while not damaging an embryo *per se*, may nevertheless result in overall harm if embryos are discarded on the basis of these tests, potentially reducing the overall pregnancy rate per stimulated IVF cycle started. That is, they may result in harm if the results are relied upon not only to prioritise embryos for transfer but also to discard them, as was observed in the instance of the premature introduction of an unvalidated culture media testing described above. Both tests, biopsy-dependent and non-invasive, have one thing in common. Considering the current uncertainties surrounding their clinical utility, they cannot be regarded as diagnostic (24, 75, 76). Non-diagnostic tests, which nevertheless may have clinical utility, can be thought of as screening tests. Indeed, the term “screening” is part of the term used to designate the currently used biopsy-dependent technique of embryo selection (PGT-S). This nuance appears to be lost on its most ardent advocates. The negative predictive value (i.e., embryo classified as normal being normal) appears very high, while the positive predictive value (i.e., embryo classified as abnormal being abnormal) is the main point of contention. Therefore, the appropriate approach to interpreting the results of these tests appears to be to use them to select embryos rather than select out and discard them. This implies that they should only be used for embryo selection and prioritisation for transfer.

The finality of discarding possibly viable embryos, which may, even rarely, produce a healthy child, should preclude these technologies from being widely used to discard embryos. Embryos deemed “abnormal” by the use of these technologies should not be discarded but should remain frozen until such time as patients no longer desire treatment or no “better” embryos are available. Under those circumstances, patients should be given a choice. Appropriate non-directive counselling should be offered, encompassing the uncertainties inherent in the use of these technologies. Various possible outcomes of an embryo classified as “abnormal” being transferred should be explored, including: the possibility of non-implantation, implantation which ultimately results in a miscarriage, a pregnancy producing a healthy child and, crucially a pregnancy that may produce a child with a disability. Patient autonomy must be respected, and the ultimate decision as to the fate of her embryos must be left to the patient. Embryos, by virtue of their reproductive potential may, under limited circumstances, be considered an extension of a patient's body (77, 78). This implies that the usual considerations of bodily autonomy must be acknowledged, and the choice to transfer an “abnormal” embryo must be available when no other embryos are obtainable. Just as it is ethically unacceptable to force someone to terminate a pregnancy, even if it is known that it will produce a child with a significant disability, it is likewise ethically questionable to decline to transfer an “abnormal” embryo, especially under the circumstances where an “abnormality” is only assumed but not conclusively proven. Needless to say, further antenatal testing and the possibility of pregnancy termination, even at late gestation, must be discussed and offered as required, which admittedly can be problematic in some jurisdictions (79).

It is worth noting that the principle of professional autonomy grants fertility specialists the discretion to make decisions based on their expertise, judgment, and ethical considerations. In the context of transferring possibly “abnormal” embryos, based on the novel selection strategies, professional autonomy might allow clinicians to refuse the transfer outside a research setting based on medical risks and the potential for the child's compromised quality of life. Ethically, this refusal can be justified by prioritizing the principle of non-maleficence.” However, it can conflict with patient autonomy, where individuals desire to proceed despite potential risks. Striking a balance requires transparent communication, considering both medical outcomes and respecting patient values and desires.

## Conclusion

Both invasive embryo selection strategies, such as PGT-A, and non-invasive ones, such as culture fluid analysis and AI-based embryo selection, may allow for the embryos with the highest reproductive potential to be transferred first. This welcome development would reduce the time to pregnancy and may also minimise the risk of miscarriage. These are worthy goals which have substantial utility, as the emotional strain of failed embryo transfers and miscarriages, as well as the costs associated with additional embryo transfer cycles, would be reduced. One must not, however, disregard the possible harms that premature widespread clinical implementation of these technologies may cause. It is essential to be cognisant of the fact that these technologies can only ever be considered screening tests, with inherent sensitivity and specificity limitations, never quite reaching 100% accuracy.

Past experiences with preimplantation genetic testing of cleavage-stage embryos and the Victorian experience with culture fluid genetic testing are a reminder that viable embryos may be discarded, thus reducing the overall chance of motherhood for some patients. Unfortunately, the current iteration of preimplantation genetic testing of blastocyst-stage embryos may be subject to similar limitations. Embryos deemed “abnormal” are routinely discarded, often without further discussion of the limitations of this technology with the patients involved. This not only results in a possible increase in the need for further stimulated IVF cycles for these patients but also impinges on their reproductive autonomy.

Therefore, we propose that ethically acceptable embryo selection strategies should have three components, all of which must be demonstrated in appropriately conducted technology evaluation studies, which should be made available for peer review and analysis. These components are:

1. An embryo with the highest chance of producing a pregnancy is transferred first, thus reducing the time to pregnancy.
2. Embryos suspected to harbour an abnormality that may produce a child with a significant disability may be transferred only rarely, under specific circumstances, where all other reproductive options have either been exhausted or are not acceptable.
3. The overall cumulative pregnancy rate from a batch of embryos derived from a stimulated cycle must not be reduced by discarding embryos that may have a normal reproductive potential.

It is likely that PGS 1.0, the currently widely used PGT-A (PGS 2.0) and the Victorian clinic's experience with the non-invasive PGS appear to have failed the third requirement. This has likely resulted in overall harm to patients, where the pregnancy rate per stimulated IVF cycle started was reduced. Discarding embryos based on unproven technologies must not be permitted, but prioritising supposedly normal embryos with the highest reproductive potential for transfer is morally acceptable. This will ensure that the overall success rate of a stimulated IVF cycle is not reduced and might result in a shorter time to pregnancy.

## Data Availability

The original contributions presented in the study are included in the article/supplementary material, further inquiries can be directed to Alex Polyakov, alex.polyakov@mivf.com.au.
